# Microparticle-Induced Activation of the Vascular Endothelium Requires Caveolin-1/Caveolae

**DOI:** 10.1371/journal.pone.0149272

**Published:** 2016-02-18

**Authors:** Allison M. Andrews, Victor Rizzo

**Affiliations:** 1 Independence Blue Cross Cardiovascular Research Center, Lewis Katz School of Medicine at Temple University, Philadelphia, Pennsylvania, United States of America; 2 Department of Anatomy and Cell Biology, Lewis Katz School of Medicine at Temple University, Philadelphia, Pennsylvania, United States of America; National Institutes of Health, UNITED STATES

## Abstract

Microparticles (MPs) are small membrane fragments shed from normal as well as activated, apoptotic or injured cells. Emerging evidence implicates MPs as a causal and/or contributing factor in altering normal vascular cell phenotype through initiation of proinflammatory signal transduction events and paracrine delivery of proteins, mRNA and miRNA. However, little is known regarding the mechanism by which MPs influence these events. Caveolae are important membrane microdomains that function as centers of signal transduction and endocytosis. Here, we tested the concept that the MP-induced pro-inflammatory phenotype shift in endothelial cells (ECs) depends on caveolae. Consistent with previous reports, MP challenge activated ECs as evidenced by upregulation of intracellular adhesion molecule-1 (ICAM-1) expression. ICAM-1 upregulation was mediated by activation of NF-κB, Poly [ADP-ribose] polymerase 1 (PARP-1) and the epidermal growth factor receptor (EGFR). This response was absent in ECs lacking caveolin-1/caveolae. To test whether caveolae-mediated endocytosis, a dynamin-2 dependent process, is a feature of the proinflammatory response, EC’s were pretreated with the dynamin-2 inhibitor dynasore. Similar to observations in cells lacking caveolin-1, inhibition of endocytosis significantly attenuated MPs effects including, EGFR phosphorylation, activation of NF-κB and upregulation of ICAM-1 expression. Thus, our results indicate that caveolae play a role in mediating the pro-inflammatory signaling pathways which lead to EC activation in response to MPs.

## Introduction

Microparticles (MPs) are small 0.1 to 1.0 μm membrane fragments shed from activated, apoptotic or injured cells. MPs circulate in the blood of healthy patients and become elevated in patients with cardiovascular diseases such as hypertension and atherosclerosis [1]. MPs are primarily shed from endothelial cells, platelets and immune cells [2] and carry proteins, mRNA and miRNA [3]. In addition, they can interact and/or be taken up in other cells and affect signaling, protein expression and cellular phenotype [4, 5]. Recent evidence suggests that MPs can play a role in inflammation [6, 7], thrombosis [8], coagulation [9], impair vasorelaxation [10] and induce oxidative stress [7, 11] thus contributing to disease progression.

MPs make up a subset of a larger classification of extracellular microvesicles, which also includes exosomes and apoptotic bodies [12]. They differ from other microvesicles in their size and mechanism of release, which is through outward pinching off of the plasma membrane. As a result of this process the protein content will largely reflect the host cell from which it was derived. MPs are typically isolated by differential centrifugation, which allows for their separation from the other microvesicle subsets [13]. A few studies have examined effects of endothelial-derived microparticles (EMPs) on other endothelial cells. These studies have demonstrated EC uptake of MPs [6], upregulation of adhesion molecules [7] and increased platelet adhesion [14]. However, the mechanism by which MPs initiate these pro-inflammatory EC responses remains incomplete. In addition, ECs are known to engage in paracrine signaling, thus making the study of EMPs important for understanding vascular signaling during pathological conditions [15].

Caveolae are 50–100 nm diameter invaginations of the plasma membrane that function as signal transduction and endocytotic centers [16]. While caveolae are expressed in several cell types including smooth muscle cells and fibroblasts [17], they are highly enriched in the endothelium where they play important roles in signaling and vascular function [18]. Studies on Caveolin-1 deficient (Cav-1-/-) mice have revealed that loss of Cav-1/caveolae provides protection against the development of several vascular diseases. For example, Cav-1-/- mice crossed with ApoE-/- mice were protected from high fat-diet induced atherosclerosis, instead forming fewer and smaller plaques than ApoE-/- mice [19]. In addition, our group recently demonstrated that Cav-1-/- mice are protected from Angiotensin II (Ang II)-induced aneurism formation and rupture [20]. Interestingly, circulating MPs are elevated in both these conditions [7, 21, 22], which suggest a role for Cav-1/caveolae in MP-induced signaling. We have therefore tested the hypothesis that MP-induced activation of the endothelium requires Cav-1/caveolae. These studies have examined this concept through the use of Cav-1 knock-out ECs as well as pharmacological inhibition of caveolae endocytosis, which is an important aspect of Cav-1/caveolae function and signaling. Our results ultimately shed light on the mechanism of MP-induced activation of the endothelium.

## Methods

### Cell Culture

Wild type (WT—c57/bl6)) and caveolin-1 knockout (Cav-1 -/- on a c57/bl6 background) Mouse Lung Endothelial Cells (MLEC) were a gift from Dr. Shampa Chatterjee (University of Pennsylvania). MLECs were harvested from mice under protocol 801630, which was approved by IACUC at University of Pennsylvania. Western blot of Cav-1 expression in each cell type is shown in S1 Fig. Cells were cultured in MCDB-131 (Sigma), 15% FBS (Benchmark) and 0.05 mg/mL gentimyacin (Cambrex Biosciences) and maintained at 37°C, 97% humidity and 5% carbon dioxide. All experiments were performed below passage 12.

### MP generation

Cells were treated with 10 ng/mL TNF-α (Sigma Aldrich) in 1% FBS for 24 hrs. Media was collected and centrifuged at 1500 g for 20 min to remove cell debris. Supernatant was either used immediately or snap froze in liquid N_2_ and stored at -80°C for later use. MPs were collected by ultracentrifugation of the supernatant at 20,000 g for 40 min followed by a second round of ultracentrifugation (20,000 g for 40min). MPs were collected by resuspension in 1% FBS and sterile filtered (0.22 μm). MP size distribution was verified using the Nanosight (NanoSight Ltd, data not shown).

### Flow Cytometry

**Fig 1:**
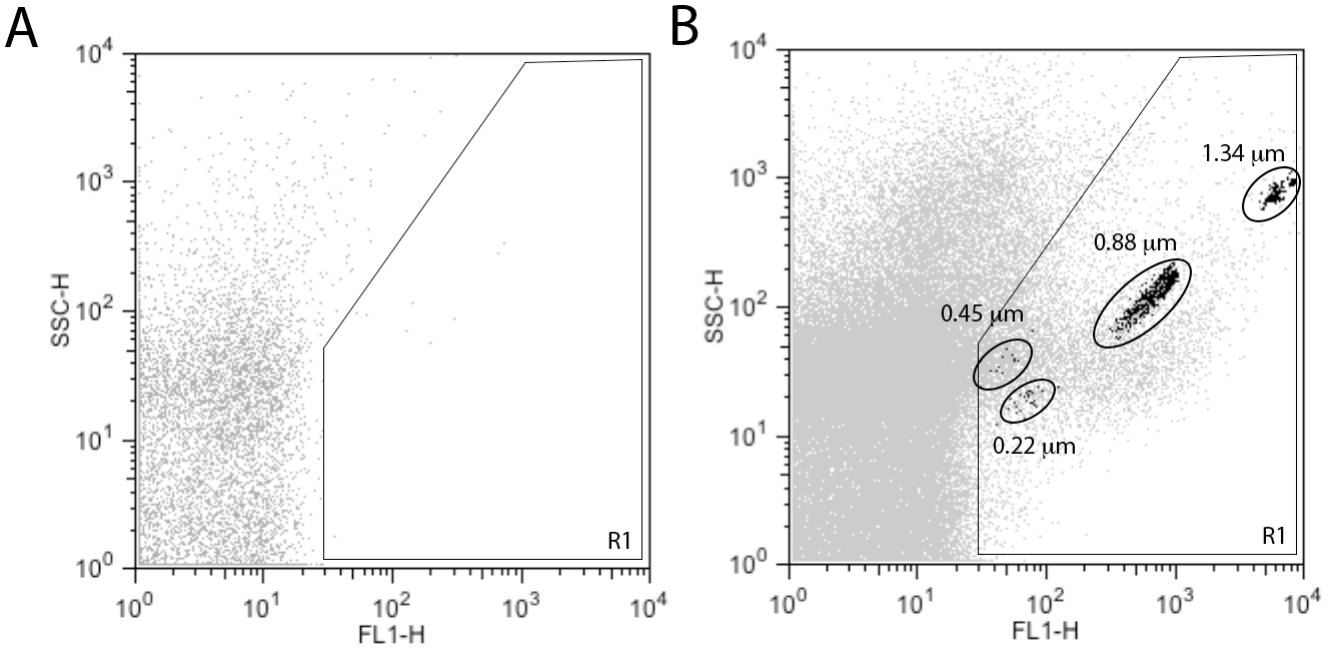
Detection of TNF-α-induced microparticles (MPs) by flow cytometry. Cultured mouse lung endothelial (MLEC) cells were treated with TNF-α (10 ng/mL) for 24 hrs. Media was collected, centrifuged and resuspended in Annexin Binding Buffer, as described in Methods. A region of interest was drawn (R1) to indicate isolated MPs from TNF-α treated cells (B, grey) as compared to unlabeled MPs (A). Size beads (0.22, 0.45, 0.88, 1.34 μm) were used to estimate MP sizes (B, black).

According to past protocols for MP isolation [7, 23, 24], MPs from thawed or freshly prepared samples were resuspended in 100 μL Annexin Binding Buffer (10mM Hepes, 140mM NaCl, 2.5mM CaCl_2_). Control samples were resuspended in annexin binding buffer without calcium. Samples were incubated with 5 μL of Annexin-FITC (BD Biosciences) for 15 min in the dark, then diluted in annexin binding buffer and analyzed on a FACScan (BD Biosciences). A region of interest based on the control sample was used to identify Annexin+ particles. Size of MPs was evaluated using fluorescent SPHERO Nano fluorescent reference beads ranging between 0.22 and 1.34 μm (Spherotech) (Fig 1). Absolute count for microparticles was determined using CountBright Counting Beads (Invitrogen) and the following formula: (Number of Annexin V+/Number of Beads) * (Bead Count of Lot/ Sample volume).

### MP Treatment

WT and Cav-1-/- cells were treated with MPs (Approx. 40,000 MPs/mL) for 0, 30 min, 1 hr, 2 hrs or 24 hrs. In some experiments, cells were incubated with 60 μM dynasore (Tocris Bioscience), 1–100 μM AG1478, or 0.1% Dimethyl sulfoxide (DMSO) for 30 minutes or 2 μM PJ34 (Enzo Life Sciences) for 1 hr prior to treatment with MPs.

### Western Blot Analysis

Cells were harvested in Mammalian Protein Extraction (MPER) lysis buffer (Thermo Scientific) with sodium vanadate (Fisher), phosphatase and protease inhibitors (Calbiochem). Proteins were separated by 5–20% SDS-PAGE and transferred to nitrocellulose membranes. Proteins of interested were detected by Western blot analysis using the following primary antibodies: ICAM-1 (polyclonal Santa Cruz Biotechnologies), β-Actin (monoclonal Sigma Aldrich), pEGFR pY1068 (polyclonal Invitrogen), EGFR (polyclonal Santa Cruz Biotechnologies), PARP-1 (polyclonal Cell Signaling), p-p65 (monoclonal Cell Signaling), p65 (polyclonal Cell Signaling), p-p50 (polyclonal Santa Cruz Biotechnologies) and p50 (polyclonal Cell Signaling).

## Results

### Caveolin-1/Caveolae mediates MP-induced increase in ICAM-1 expression via activation of NF-κB and PARP-1

**Fig 2:**
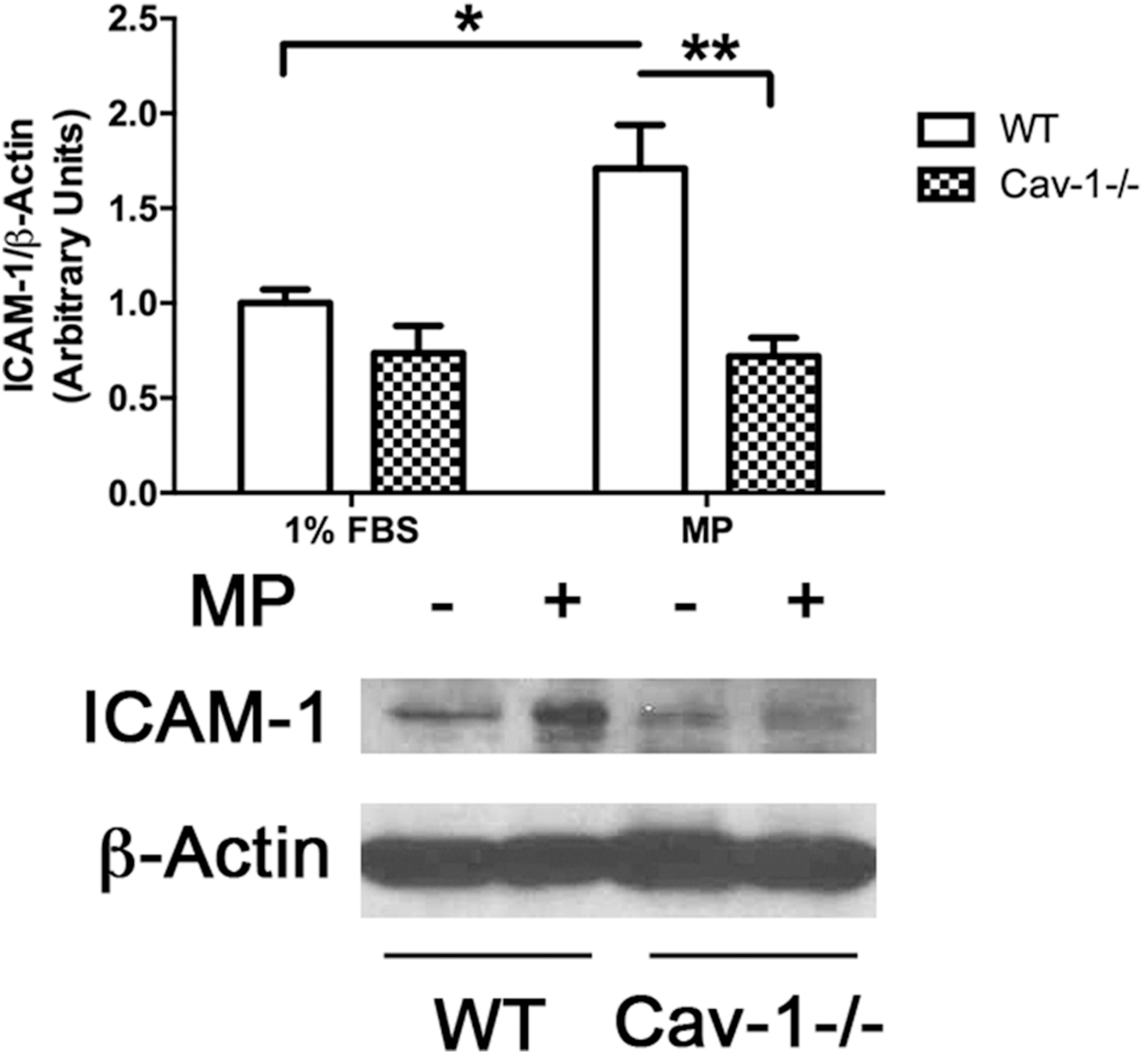
MP-induced ICAM-1 expression requires caveolin-1/caveolae. WT and Cav1-/- MLEC’s were treated with MPs (Approx 40,000 MPs/mL) for 24 hrs. Cells were collected, homogenized, lysed and total cellular protein separated by 5–15% SDS-PAGE followed by Western blotting to detect ICAM-1 and β-Actin. Densitometric quantification showed ~2 fold increase in ICAM-1 expression in WT cells in response to MPs. In Cav-1-/- cells, basal expression of ICAM-1 was lower than that detected in WT cells and it remained unchanged in response to MPs. (Avg ± SEM two-way ANOVA and bonferroni’s post hoc analysis, n = 8, *p<0.05, **p<0.01).

To examine the effects of MPs on the endothelium, MLECs were treated with MPs and cell lysates harvested and examined using Western blot analysis. MPs induced an increase in ICAM-1 protein expression by 2-fold in response to MPs after 24 hrs. Given the role of caveolae as signaling microdomains in the endothelium, we next tested whether caveolae organelles also participated in MP-induced signaling pathways that govern adhesion molecule expression. Here, we utilized endothelial cells derived from caveolin-1 knockout mice. Interestingly, baseline expression of ICAM-1 was reduced by 25% in Cav-1-/- endothelial cells compared to their WT counterpart. In addition, in response to 24hr exposure to MPs, Cav-1-/- EC’s did not upregulate ICAM-1 expression (Fig 2).

**Fig 3:**
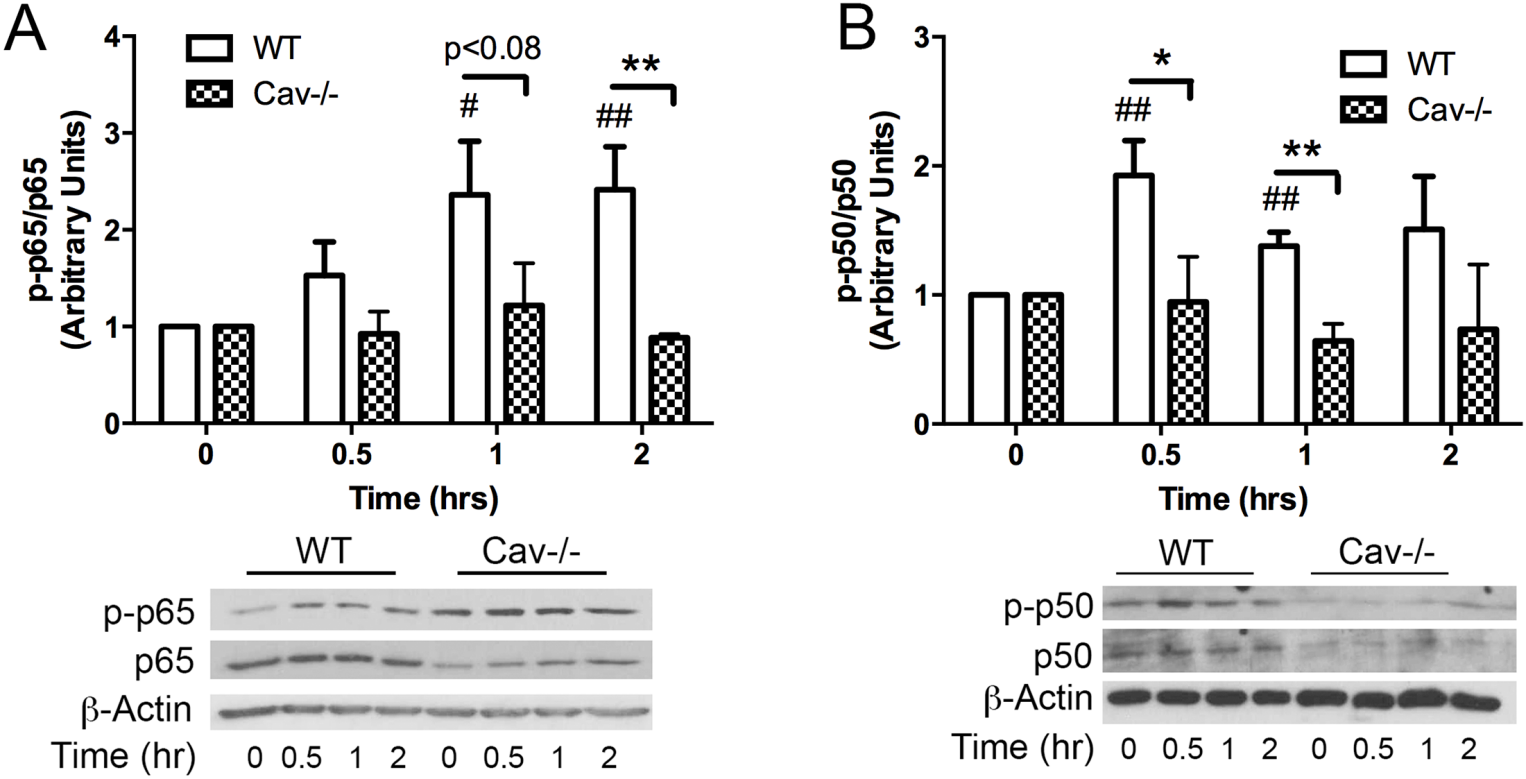
MPs induce phosphorylation of NF-κB is absent in Cav-1-/- MLECs. WT and Cav-1-/- cell cultures were treated with MPs (Approx 40,000 MPs/mL) for indicated times (0, 0.5, 1, 2 hrs) and processed for Western blot analysis. WT cells showed increased phosphorylation of both p65 (A) and p50 (B) in response to MPs. However, MP’s did not induce phosphorylation of either subunit in Cav-1-/- cells. All values are normalized to t = 0 for each cell type respectively (Avg ± SEM two-way ANOVA and bonferoni post-hoc analysis A. p-p65/p65: WT and Cav-1-/- n = 4 each, B. p-p50/p50: WT n = 6 and Cav1-/- n = 4 for all time points except one which was excluded from each group at t = 0.5 hr based on Grubb’s test for outliers p<0.05. # denotes comparison with WT t = 0, #p<0.05, ##p<0.01, * denotes comparisons between WT and Cav-1-/- cells *p<0.05, **p<0.01.)

To test the involvement of NF-κB in MP induced signaling, cells were treated with MPs for up to 2hrs and processed for Western blot analysis to detect expression and phosphorylation status of the NF-κB subunits p65 and p50. WT MLECs showed increased phosphorylation of both p65 and p50 over the time course of study in response to MPs (Fig 3A). In Cav-1-/- MLECs, basal levels of both p65 and p50 were somewhat reduced compared to WT. While enhanced baseline phosphorylation of p65 was also noted in Cav-1-/- cells, MPs failed to enhance p65 or p50 levels beyond baseline at all observed time points (Fig 3B).

**Fig 4:**
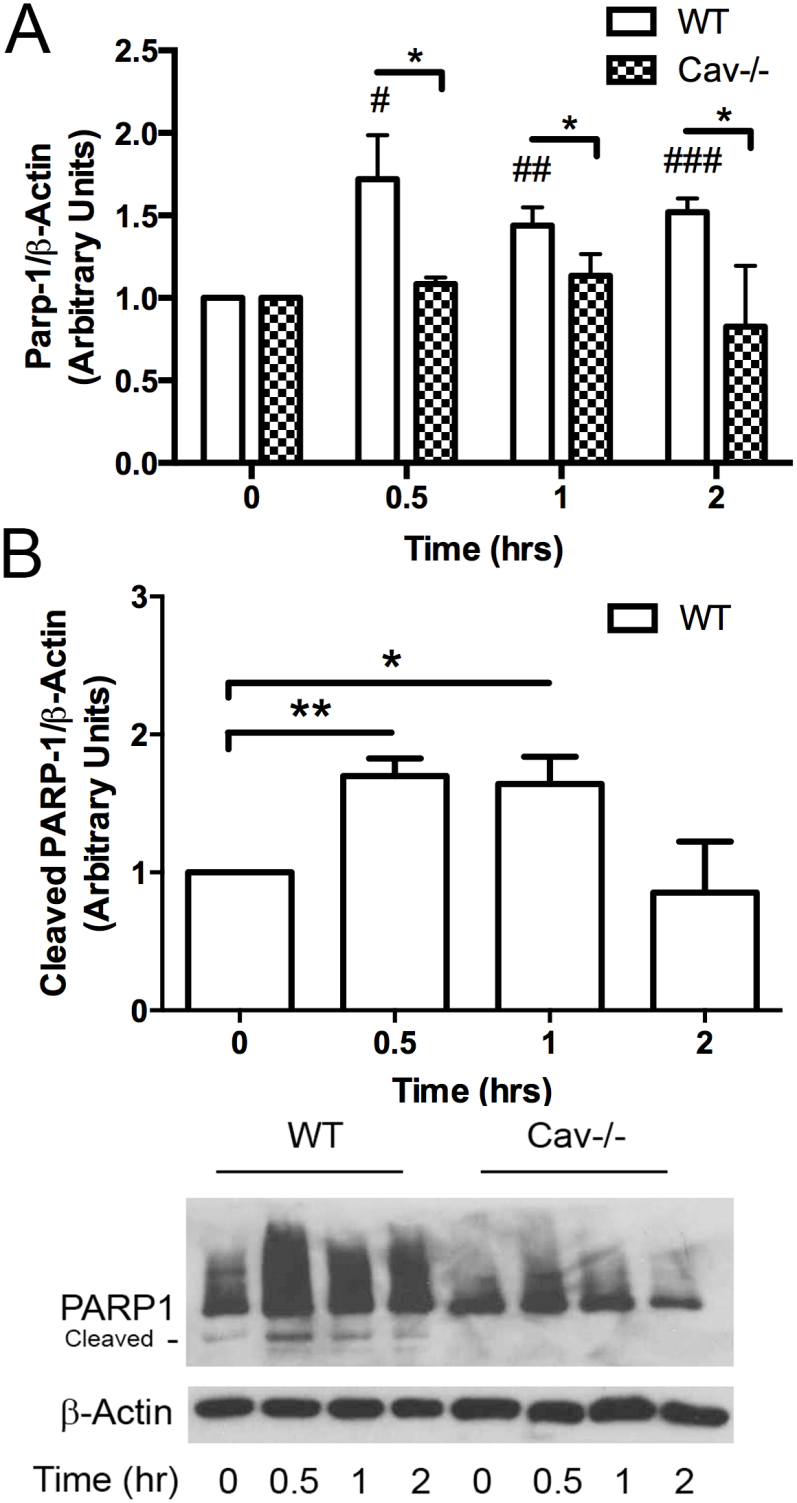
Loss of caveolin-1/caveolae abolish MP-induced production of cleaved and uncleaved PARP-1. WT and Cav-1-/- MLECs were treated with MPs for indicated times (0, 0.5, 1, 2 hrs). Proteins from whole cell lysates were separated using SDS-page (5–15%, transferred to nitrocellulose membranes, which were probed for PARP-1. MPs induced a ~ 2 fold increase in cleaved and uncleaved PARP-1 in WT cells. Basal expression of PARP-1 was reduced in Cav-1-/- cells. PARP-1 expression and cleavage pattern remained unchanged following treatment with MPs. All values are normalized to t = 0 for each cell type respectively (Avg ± SEM Two-way ANOVA and bonferroni post hoc analysis *p<0.05 A. PARP-1: WT and Cav1-/- n = 5, B. Cleaved PARP-1: Cav1-/- n = 5 except t = 2 hr n = 4, cleaved PARP-1: WT n = 4 for all time points except one was excluded at t = 2hrs using Grubbs test for outliers p<0.05; # denotes comparisons to WT t = 0, #p<0.05, ##p<0.01, ###p<0.001, * denotes comparisons between WT and Cav-1-/- cells, *p<0.05).

Next, we examined whether PARP-1 was activated (as determined by increased PARP-1 expression) in response to MPs. WT MLECs showed clear activation of PARP-1 in response to MPs over the course of 2 hrs (Fig 4A). Cav-1-/- MLECs had decreased basal levels of PARP-1 and expression did not change in response to MPs (Fig 4A). In addition to PARP-1 activity, we detected changes in the presence of cleaved PARP-1. WT MLECs showed ~ 2 fold increase in PARP-1 cleaved fragments in response to MPs while Cav-1-/- MLECs had no detectable PARP-1 fragments at basal levels or in response to MPs (Fig 4B).

### MP-induced increase in ICAM-1 expression is dependent on EGFR activation

**Fig 5:**
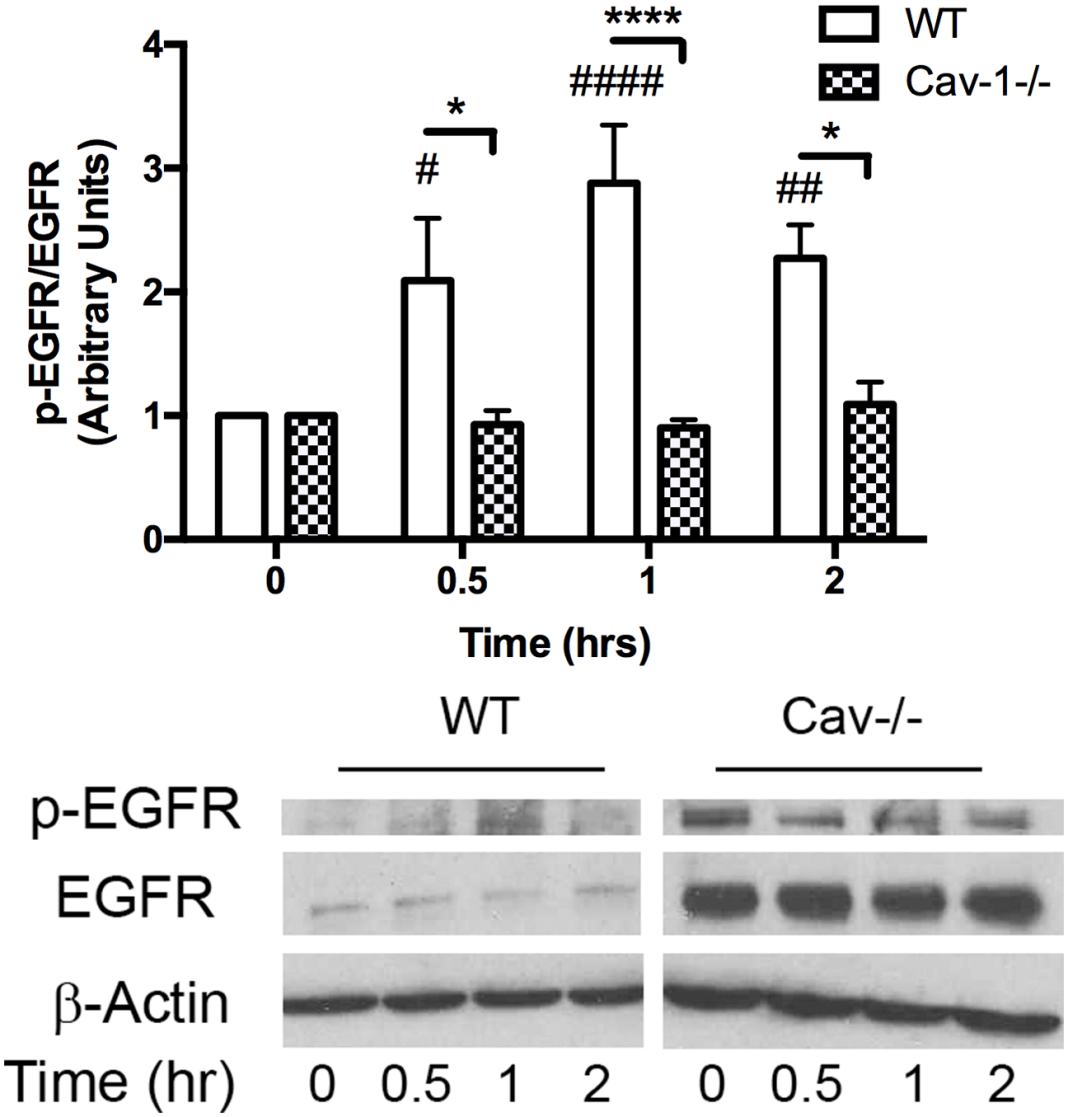
Caveolin-1/Caveolae mediates MP-induced activation of EGFR. WT and Cav-1-/- MLECs were treated with MPs for 0, 30, 1hr, and 2 hrs and processed for Western blotting. WT cells showed an increase in EGFR phosphorylation that peaked at ~3 fold after 1 hr of MP treatment. Cav-1-/- cells had increased expression of pEGFR and EGFR, however, EGFR phosphorylation was unchanged in response to MPs. All values are normalized to t = 0 for each cell type respectively (Avg ± SEM two-way ANOVA and bonferroni post hoc analysis, n = 5 each, # denotes comparisons to WT t = 0, #p<0.05, ##p<0.01, ###p<0.001, * denotes comparisons between WT and Cav-1-/- cells, *p<0.05, ***p<0.001).

**Fig 6:**
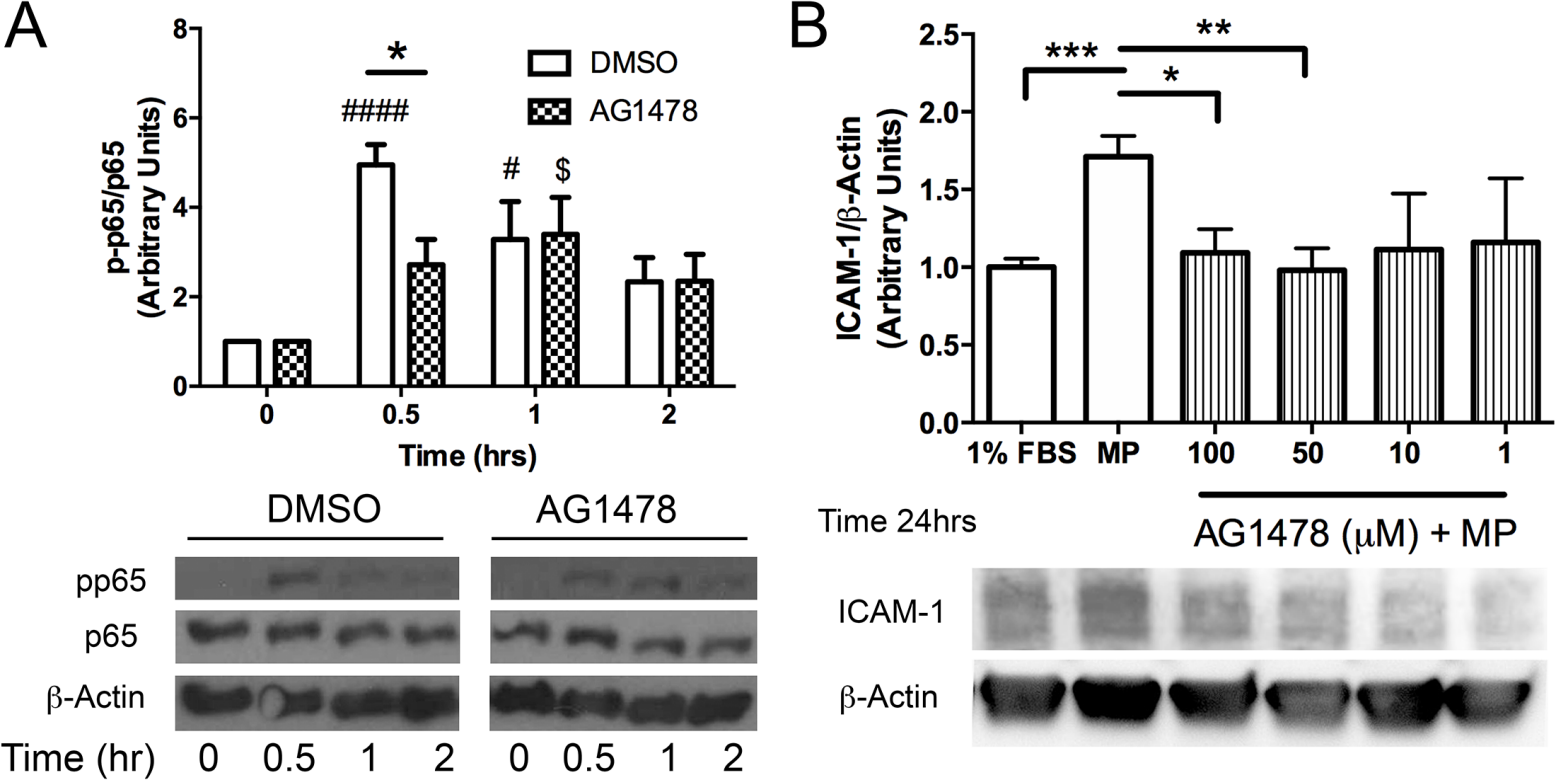
Inhibition of EGFR activation attenuates MP-induced phosphorylation of p65 and blocks upregulation of ICAM-1. WT cells were incubated with DMSO or EGFR inhibitor AG1478 (1–100 μm) for 30 min prior to treatment with MPs (Approx 40,000 MPs/mL) for time points indicated (0, 0.5, 1, 2, 24 hrs). Cells were then harvested, separated by SDS-PAGE and blotted for the indicated proteins. Inhibition of EGFR with AG1478 blocked phosphorylation of p65 (A) and inhibited upregulation of ICAM-1 in a concentration dependent manner (B). (Avg ± SEM Two-way ANOVA and bonferroni post-hoc analysis for p65 and dunnetts post-hoc test for ICAM-1 A. DMSO: n = 6 AG1478: n = 5 # denotes comparison with DMSO t = 0, #p<0.05, ####p<0.0001, * denotes comparison between DMSO and AG1478 treated cells, *p<0.05, $ denotes comparison with AG1478 at t = 0, $p<0.05. B. ICAM-1 expression: 1% FBS and MP n = 9, AG1478 n = 4 for each concentration *p<0.05, ** p<0.01, ***p<0.001)

As a potential upstream mediator of an NF-κB /PARP-1 pathway, we examined the effect of MPs on EGFR activation. The data showed that MPs caused a rise in EGFR phosphorylation, which peaked at 1hr after treatment (Fig 5). Cav-1-/- MLECs, while displaying an enhanced baseline level of the receptor, showed no change in receptor phosphorylation status in response to MPs (Fig 5). Although the EGFR had been implicated in MP induced activation of the endothelium, the consequences of receptor activation have not been determined. Here, we examined whether inhibiting EGFR activation using AG1478 would block downstream NF-κB phosphorylation and the induction of ICAM-1 expression. We observed a decrease in phosphorylation of the NF-κB p65 subunit at 30 min after stimulation with MPs (Fig 6A) as well as no change in the expression of ICAM-1 at 24 hrs in cells pretreated with AG1478 (Fig 6B).

### PARP-1 inhibition blocks MP-induced PARP-1 activation and upregulation of ICAM-1

**Fig 7:**
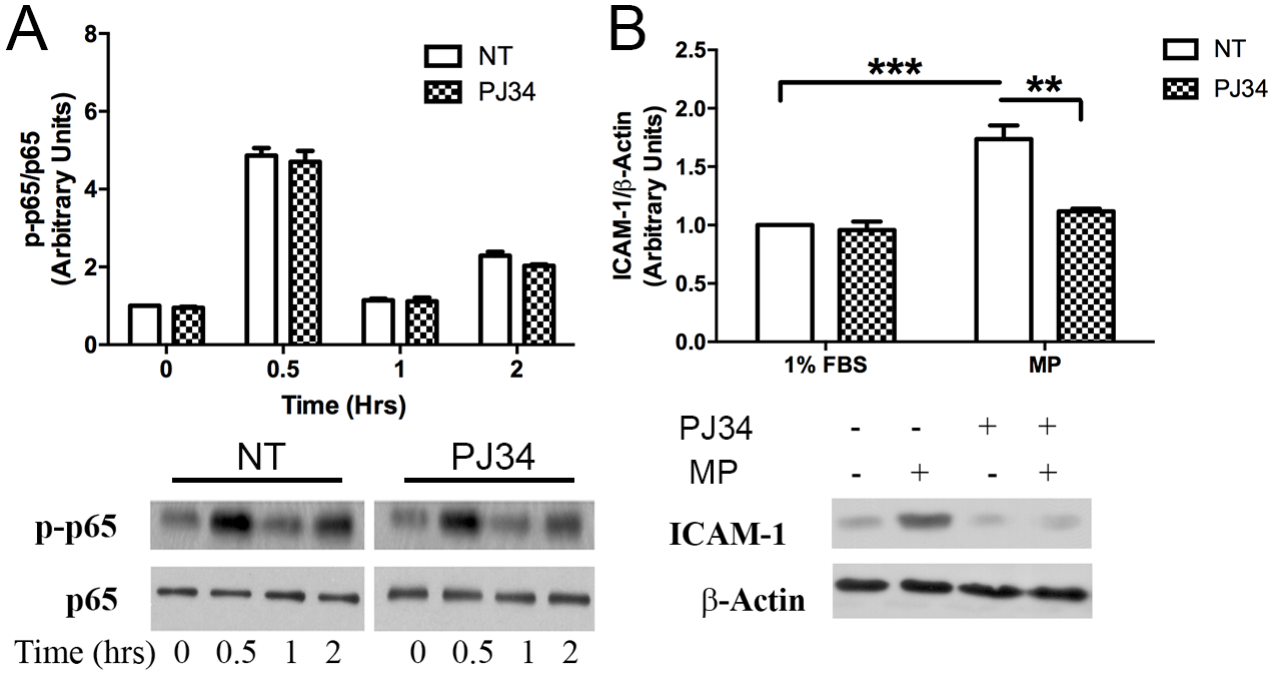
PARP-1 inhibition blocks upregulation of ICAM-1. MLECs were pretreated with the PARP-1 inhibitor PJ34 2 μM for 1 hr prior to incubation with MPs for the indicated time points (0, 0.5, 1, 2, 24 hrs). Cells were then harvested, separated by SDS-Page and blotted for the proteins indicated. The PARP-1 inhibitor PJ34 clearly blocked MP induced upregulation of ICAM-1 (A). In addition, PARP-1 inhibition did not block NF-κB phosphorylation indicating that NF-κB activation is upstream of PARP-1 (B) (Avg ± SEM two-way ANOVA and bonferroni post hoc test n = 3 each condition).

We have shown that MPs induce PARP-1 activation and cleavage (Fig 4A and 4B). We next sought to determine where PARP-1 exerts its effects in the MP-induced signaling pathway, which leads to upregulation of ICAM-1. We utilized the PARP-1 inhibitor PJ34, which has previously been shown to block PARP-1 activation in endothelial cells [25]. PJ34 pretreatment effectively blocked MP-induced PARP-1 activation and cleavage (S2 Fig). In addition, PARP-1 inhibition did not affect NF-κB phosphorylation but was able to attenuate ICAM-1 upregulation (Fig 7).

### Pharmacological inhibition of caveolae endocytosis attenuates MP-induced increase in ICAM-1 expression

**Fig 8:**
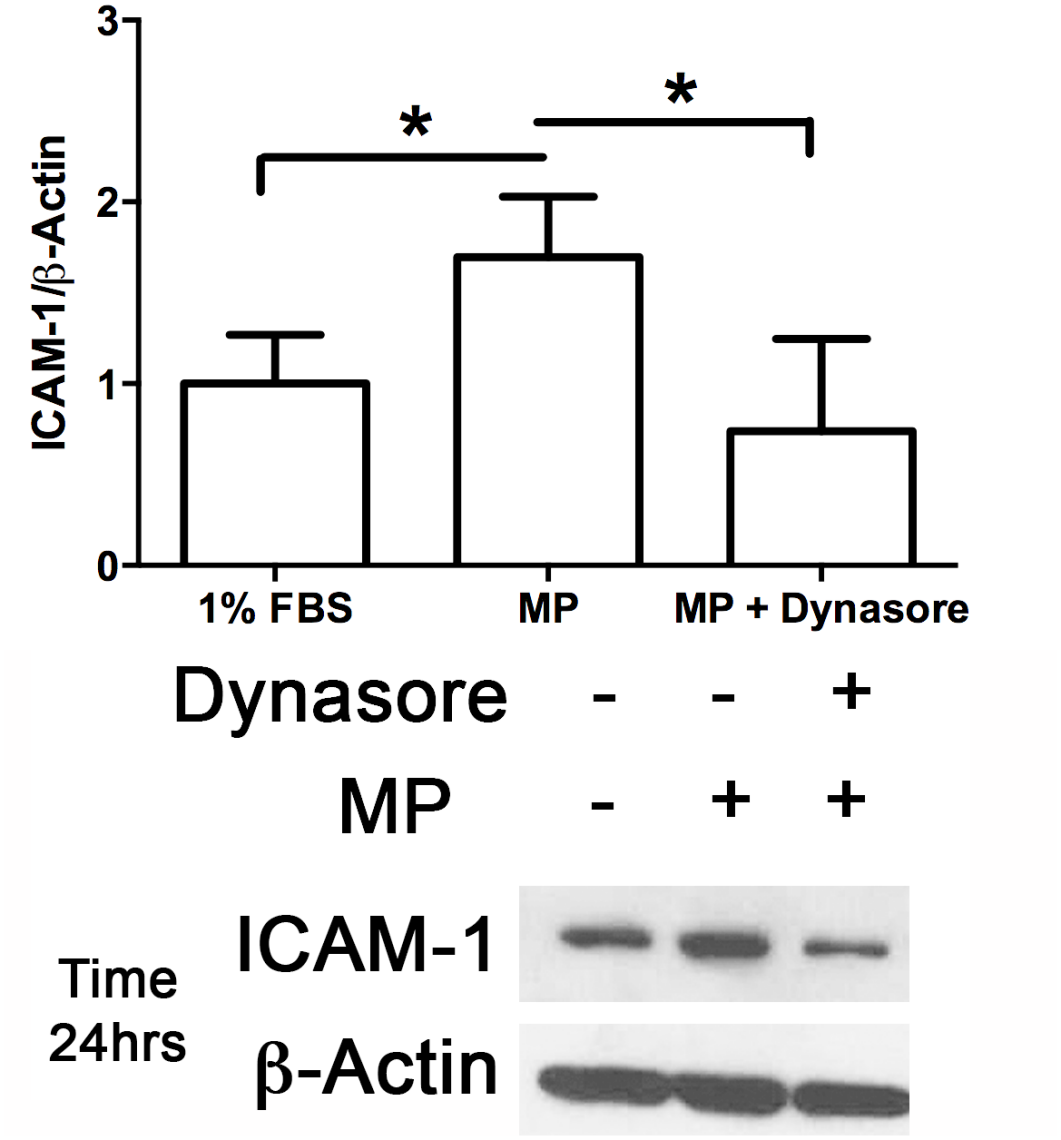
Inhibition of Caveolae endocytosis blocks upregulation of ICAM-1 expression. MLECs were pretreated with either 60 μM Dynasore or 0.1% DMSO for 30 min prior to incubation with MPs (Approx 40,000 MPs/mL) for the time points indicated (0, 0.5, 1, 2, 24 hrs). Cells were then harvested, separated with SDS-PAGE and blotted for ICAM-1 and β-Actin. MP-induced upregulation of ICAM-1 protein expression was significantly inhibited in cell treated with the dynamin-2 inhibitor dynasore. (Avg ± SEM two-tailed t-test, n = 4 each condition, *p<0.05).

**Fig 9:**
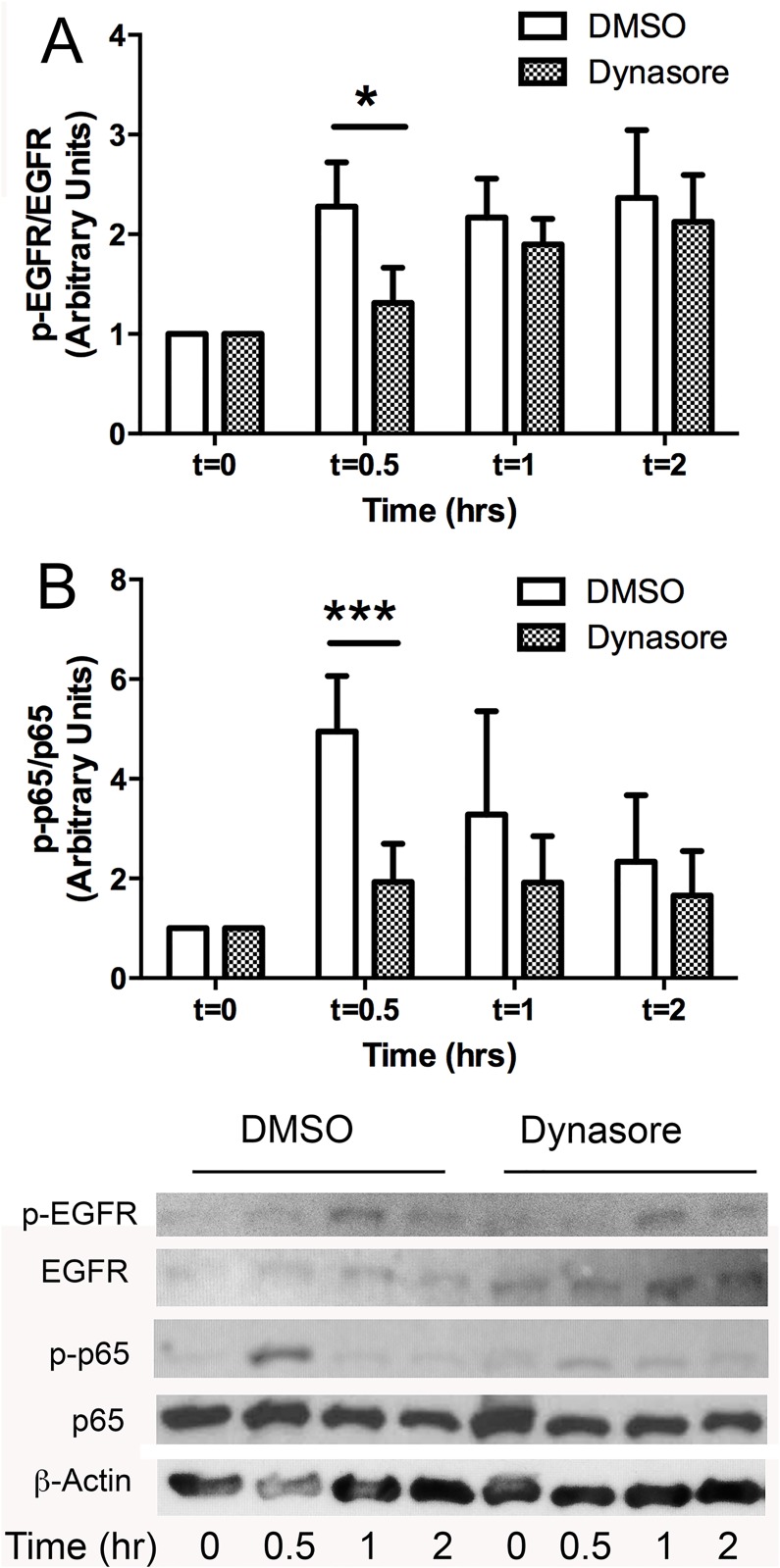
Caveolae endocytosis inhibition attenuates MP-induced phosphorylation of p65 and EGFR. MLECs were pretreated with 60 μM Dynasore or 0.1% DMSO for 30 min prior to incubation with MPs for indicated time points (0, 0.5, 1, 2 hrs). Cells were then harvested, separated by SDS-Page and blotted for the proteins indicated. Inhibition of caveolae endocytosis attenuated MP-induced phosphorylation of p65 (A) and EGFR (B) at 30 min. (Avg ± SEM two-way ANOVA and bonferroni post hoc analysis for p65 and Kruskal-Wallis test **p<0.01 for EGFR A. p65: n = 6 dmso n = 4 dynasore # denotes comparison with DMSO t = 0, ###p<0.001 B. n = 4 for each condition *p<0.05).

To determine whether the MP-induced increase in ICAM-1 expression requires caveolae-mediated endocytosis, we pretreated WT endothelial cells with dynasore, a dynamin-2 inhibitor. Inhibition of caveolae endocytosis blocked the MP-induced increase in ICAM-1 expression (Fig 8). To determine the involvement of caveolae endocytosis in MP-induced signaling we also examined the effect of dynasore on p65 and EGFR phosphorylation. Inhibition of caveolae endocytosis attenuated MP-induced p65 phosphorylation through all observation periods and EGFR phosphorylation at 30 min but not at later time points (Fig 9A and 9B).

## Discussion

The major finding of this study is that MPs induce activation of the endothelium through a mechanism requiring caveolin-1/caveolae endocytosis.

A number of studies have shown that in response to MPs, the endothelium increases expression of adhesion molecules *in vitro* [6, 7, 26, 27] and *in vivo* [7], which causes increased rolling, and adhesion of leukocytes [26]. Increased adhesion molecule expression is a fundamental step in the progression of a number of cardiovascular diseases such as atherosclerosis [28]. MPs have been shown to interact with the endothelium surface and in some cases are internalized [14]. However, a “MP-receptor” has yet to be determined and the mechanisms by which MPs activate the endothelium remain unclear.

By virtue of their signaling properties, lipid rafts and more specifically caveolae, play important roles in many aspects of endothelial cell function [16]. To date, there are very few studies, which have investigated the relationship between MPs and these membrane microdomains. Related to MP production, one study showed that Ang II-induced MP release from the endothelium could be blocked by pretreating cells with methy–β-cyclodextrin, a compound which disrupts raft integrity. In the present study, we asked whether caveolin-1/caveolae could influence MP-induced responses in the endothelium.

Upregulation of adhesion molecules by MPs has been reported in several systems and involve upstream activation of the NF-κB transcription factor [27, 29–33]. Here we report that MP-induced activation of NF-κB in MLECs (Fig 3A) followed the same timeline for activation as reported in other cell types [27] where phosphorylation of p65 occurs by 30 min and remains elevated through 2hr of exposure to MPs. In our studies we found that the loss of caveolin-1/caveolae prevented MP-induced phosphorylation of both NF-κB subunits (p65 and p50) (Fig 3A and 3B). This finding is the first to show an association between Cav-1/caveolae and MP signaling and is consistent with a role for caveolae in NF-κB activation by proinflammatory agents [34]. Although the loss of caveolin-1 prevented activation of NF-κB, we observed that the basal expression of both p-p65 and p-65 were upregulated (Fig 2B) while basal expression of ICAM-1 was downregulated in caveolin-1 null cells (Fig 2). These findings indicate that Cav-1 expression is required for the conversion of activated NF-κB to the transcription of ICAM-1. In addition, increased basal p65 expression suggests that caveolin-1 or another caveolae-associated protein may be involved in regulating p65 expression. The mechanism for this is unclear, however, Tiruppathi et al. (2008) have reported that the Cav-1 gene sequence contains 2 NF-κB binding domains that transcriptionally regulate Cav-1 [35] making it feasible that Cav-1 is in turn involved in p65 regulation.

PARP-1 is a very versatile metabolite of nicotinamide adenine dinucleotide (NAD) and is known for its involvement in transcription, DNA damage and repair and apoptosis [36]. Loss of PARP-1 has been shown to prevent NF-κB activation in response to H2O2 [37] and TNF-α [38] as well as the upregulation of ICAM-1 [38]. PARP-1 is thought to mediate nucleus translocation of NFκB [38, 39], however, PARP-1 is not required for upregulation of all adhesion molecules such as VCAM-1 [38]. Here we evaluated the role of PARP-1 as a signaling mediator in relationship to MP and caveolae mediated pathways. We found increased PARP-1 expression in WT cells in response to MPs (Fig 4A). Interestingly, Cav-1-/- cells showed reduced basal levels of PARP-1, which did not change in response to MPs (Fig 4A). In addition to increased PARP-1 expression, we also found an increase in PARP-1 fragments (Fig 4C). To evaluate the role of PARP-1 in MP-induced activation of the endothelium we utilized a PARP-1 inhibitor. The PARP-1 inhibitor PJ34 effectively blocked the MP-induced PARP-1 activation and cleavage (S2 Fig) and attenuated the MP-induced upregulation of ICAM-1. However, it did not affect NF-κB phosphorylation (Fig 7), which is supported by recent studies on the involvement of PARP-1 in NF–κB signaling and transcriptional activation [40]. PARP-1 fragments are widely accepted to be biomarkers of protease activity involved in cellular apoptosis pathways [41]. The induction of PARP fragments suggests the initiation of pro-apoptotic pathways. MPs have been shown to induce cleavage of caspase 3, another indicator of activation of apoptosis pathways [42]. Our data indicates that Cav-1-/- cells did not produce PARP-1 fragments (Fig 4) suggesting a protective effect against MP-induced apoptosis. This is in contrast to findings by Xu et al. (2014) who found that Cav-1 knock-down in gastric cancer cells had higher levels of cleaved PARP-1 in response to TNF-α-related apoptosis-inducing ligand (TRAIL) compared to cells containing Cav-1 [43]. Previous studies have found Cav-1 linked to cleavage of PARP-1 through the estrogen receptor ERα. ERα becomes dissociated from caveolin-1 upon de-palmitoylation and in turn activates p38 and PARP cleavage [44]. Other hormone receptors also rely heavily on palmitoylation and could provide the mechanism for the involvement of caveolin-1 in the generation of PARP fragments. One possible candidate is EGFR, which has been reported to be involved in MP-induced activation of the endothelium [7].

EGFR is expressed in the endothelium and involved in regulating endothelial cell functional phenotype [45]. In addition, recent reports have shown that inhibiting EGFR activation with gefitinib prevented MP-induced upregulation of VCAM-1 in the endothelium [7]. Examination of EGFR phosphorylation showed increased activity in WT MLECs in response to MPs within 30 min that was sustained over the 2 hr observation period (Fig 5). EGFR activation has been linked to both PI3K/Akt and p38 activation which results in downstream activation of NF-κB [46]. We found that EGFR inhibition with AG1478 blocked NF-κB activation (Fig 6A) and upregulation of ICAM-1 (Fig 6B) supporting previous reports [7].

A number of reports demonstrate a link between caveolin-1 and EGFR transactivation [47–49] and that loss of caveolin-1 prevents EGFR phosphorylation in response to TGF-β [49] and tumor necrosis factor-related apoptosis-inducing ligand (TRAIL) [43]. In addition, EGFR has been reported to co-localize with caveolin-1 [50–53]. However, whether Cav-1 is a positive or negative regulator of EGFR seems to be cell type dependent [54]. In the present study, we found that Cav-1-/- cells had increased baseline levels of pEGFR and EGFR expression, which remained unchanged in response to MPs (Fig 5) supporting previous reports of caveolin-1 involvement in EGFR activation. Additionally, some reports indicate an inverse relationship between caveolin expression and either EGFR [55] or phosphorylation of EGFR [54, 56], which could explain the difference in EGFR expression between WT and Cav-1-/- cells.

EGFR is internalized through both clathrin dependent [57] and independent pathways [58], which can depend on the ligand concentration and have consequences regarding the intended receptor fate (recycled vs. degradation) [59]. Both phosphorylation of EGFR and Cav-1 have been shown to occur simultaneously in response to TRAIL [43] and caveolin-1 phosphorylation regulates caveolae endocytosis [60]. Dynamin-2 is required for caveolae “pinching” off from the membrane [61] and dynamin-2 inhibitors are commonly used to block caveolae endocytosis [61, 62]. Here, we found that inhibition of caveolae endocytosis prevented MP-induced upregulation of ICAM-1 (Fig 8) and attenuated NF-κB activation (Fig 9B) as well as EGFR phosphorylation (Fig 9A) and identifies caveolae endocytosis as an important process in MP-induced signaling. We recognize that clathrin-mediated endocytosis is also dynamin-dependent [63]. However, caveolae constitute 50–70% of the surface of endothelium [64] and the lung endothelium specifically contain very few clathrin-coated pits [65] which makes the use of dynasore specific for blocking caveolae endocytosis in our experiments.

Our studies have demonstrated a role for caveolin/caveolae endocytosis in the mechanism of MP-induced activation of the endothelium. In our model, we have utilized MPs derived from microvascular endothelial cells. However, it is important to note that the composition of MPs depend on the type of endothelial cell (renal and brain microvascular (MiVEC) and coronary macrovascular) from which they were derived [21]. We are not aware of any studies that have examined whether MPs derived from ECs of different vascular linages induce unique effects in EC signaling and activation. However, ECs of the vasculature can have different phenotypes, protein and gene expression profiles. For instance, capillaries in the lung, heart and skeletal muscle are highly enriched in caveolae [65, 66], while the brain endothelium has a reduced number of caveolae [66]. Therefore, the response of the endothelium to MPs is likely dependent on the vascular linage. However, this is beyond the scope of this work but warrants future study in order to fully understand the effects of MPs on the endothelium.

**Fig 10:**
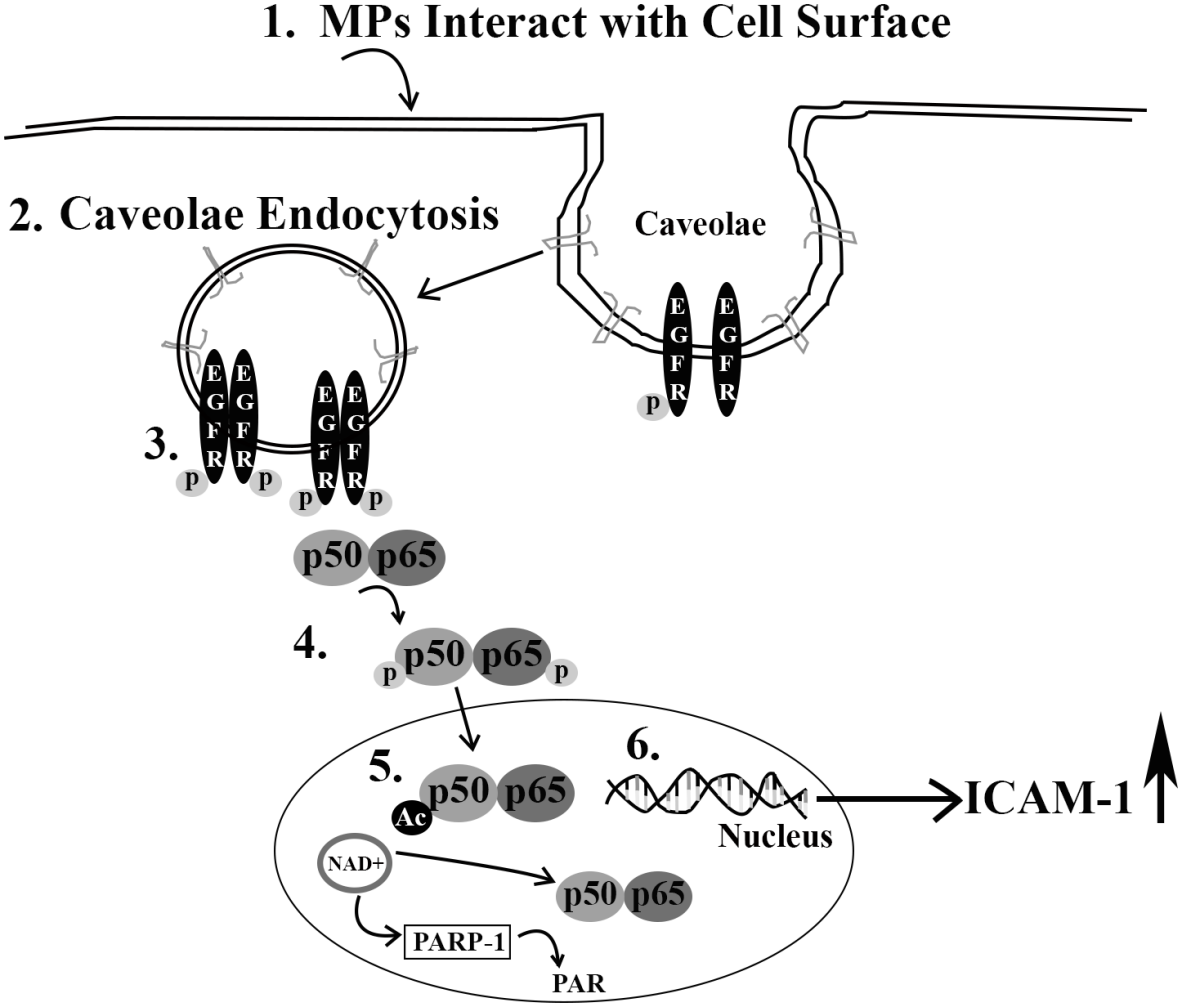
Proposed Mechanism of MP-induced activation of the endothelium. Based on the studies presented here we propose the following mechanism for MP-induced activation of the endothelium. 1. MPs interact with the cell surface and induce the 2. EGFR phosphorylation and endocytosis of caveolae 3. EGFR is further phosphorylated followed by 4. phosphorylation of NF-κB subunits p65 and p50. After NF-κB translocation to the nucleus, ICAM-1 transcription is initiated which is dependent on PARP-1 activation and PARP-1 generation.

Based on our results, we propose the following mechanism for MP-induced activation of the endothelium (Fig 10). Following MP interaction with the endothelial surface, EGFR becomes phosphorylated and is endocytosed via a caveolae-dependent mechanism. This leads to phosphorylation of NF-κB and increased PARP-1 activity and cleavage. Activation of NF-κB ultimately leads to transcriptional upregulation of ICAM-1, which is PARP-1 dependent. Collectively, our results identify a role for caveolin-1/caveolae in the propagation of signaling events involved in MP-induced activation of the endothelium. These findings may further explain the cardiovascular protective effects seen in the Cav-1 -/- mouse.

## Supporting Information

S1 Fig Cav-1 expression in WT and Cav-1-/- MLECs. Cell lysates were harvested from 3 samples for each WT and Cav-1-/- MLECs. Lysates were separated by SDS-Page and blotted for the proteins indicated. Western blots demonstrate the lack of Cav-1 expression in the Cav-1-/- MLECs.
(TIF)

S2 Fig Representative Western blot of PARP-1 inhibition by PJ34. WT MLECs were pretreated with the 2 μM of PJ34 for 1 hr prior to treatment with MPs. Cells lysates were harvested, separated SDS-Page and blotted for the proteins indicated. Western blots demonstrate inhibition by PJ34 of the MP-induced PARP-1 activation and cleavage.
(TIF)
