# Supplementary figures and images for: Microparticle-Induced Activation of the Vascular Endothelium Requires Caveolin-1/Caveolae
Source: PLoS One. 2016 Feb 18;11(2):e0149272. doi: 10.1371/journal.pone.0149272 (PMC4758735; doi:10.1371/journal.pone.0149272)

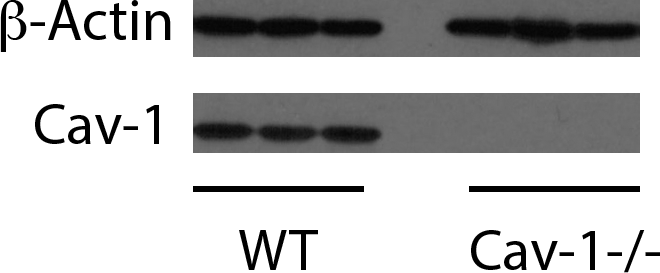

Supplement: S1 Fig — Cell lysates were harvested from 3 samples for each WT and Cav-1-/- MLECs. Lysates were separated by SDS-Page and blotted for the proteins indicated. Western blots demonstrate the lack of Cav-1 expression in the Cav-1-/- MLECs. (TIF) [file pone.0149272.s001.tif]

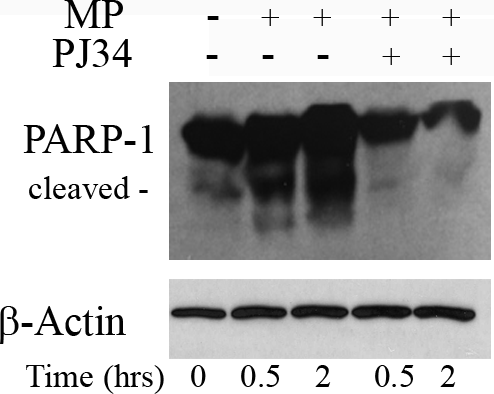

Supplement: S2 Fig — WT MLECs were pretreated with the 2 μM of PJ34 for 1 hr prior to treatment with MPs. Cells lysates were harvested, separated SDS-Page and blotted for the proteins indicated. Western blots demonstrate inhibition by PJ34 of the MP-induced PARP-1 activation and cleavage. (TIF) [file pone.0149272.s002.tif]
